# Liraglutide Activates Type 2 Deiodinase and Enhances β3-Adrenergic-Induced Thermogenesis in Mouse Adipose Tissue

**DOI:** 10.3389/fendo.2021.803363

**Published:** 2022-01-04

**Authors:** Fernanda C. B. Oliveira, Eduarda J. Bauer, Carolina M. Ribeiro, Sidney A. Pereira, Bruna T. S. Beserra, Simone M. Wajner, Ana L. Maia, Francisco A. R. Neves, Michella S. Coelho, Angelica A. Amato

**Affiliations:** ^1^ Laboratory of Molecular Pharmacology, School of Health Sciences, University of Brasilia, Brasilia, Brazil; ^2^ Endocrine Division, Hospital de Clinicas de Porto Alegre, Federal University of Rio Grande do Sul, Porto Alegre, Brazil

**Keywords:** GLP-1 receptor agonist, liraglutide, adipose tissue, β3-adrenergic stimulation, type 2 deiodinase

## Abstract

**Aims:**

Liraglutide is a long-acting glucagon-like peptide 1 (GLP-1) receptor agonist used as an anti-hyperglycemic agent in type 2 diabetes treatment and recently approved for obesity management. Weight loss is attributed to appetite suppression, but therapy may also increase energy expenditure. To further investigate the effect of GLP-1 signaling in thermogenic fat, we assessed adipose tissue oxygen consumption and type 2 deiodinase (D2) activity in mice treated with liraglutide, both basally and after β3-adrenergic treatment.

**Methods:**

Male C57BL/6J mice were randomly assigned to receive liraglutide (400 μg/kg, n=12) or vehicle (n=12). After 16 days, mice in each group were co-treated with the selective β3-adrenergic agonist CL316,243 (1 mg/kg, n=6) or vehicle (n=6) for 5 days. Adipose tissue depots were assessed for gene and protein expression, oxygen consumption, and D2 activity.

**Results:**

Liraglutide increased interscapular brown adipose tissue (iBAT) oxygen consumption and enhanced β3-adrenergic-induced oxygen consumption in iBAT and inguinal white adipose tissue (ingWAT). These effects were accompanied by upregulation of UCP-1 protein levels in iBAT and ingWAT. Notably, liraglutide increased D2 activity without significantly upregulating its mRNA levels in iBAT and exhibited additive effects to β3-adrenergic stimulation in inducing D2 activity in ingWAT.

**Conclusions:**

Liraglutide exhibits additive effects to those of β3-adrenergic stimulation in thermogenic fat and increases D2 activity in BAT, implying that it may activate this adipose tissue depot by increasing intracellular thyroid activation, adding to the currently known mechanisms of GLP-1A-induced weight loss.

## Introduction

Glucagon-like peptide 1 (GLP-1) is an incretin hormone with pleiotropic physiological effects, many of which occur at key sites of energy balance control and favorably affect metabolic homeostasis. Supraphysiological GLP-1 signaling by GLP-1A (GLP-1A) is exploited in the treatment of several metabolic disorders, such as type 2 diabetes, obesity, non-alcoholic fatty liver disease, and cardiovascular disease (1). GLP-1A are currently approved for hyperglycemia treatment in type 2 diabetes due to their action to stimulate glucose-dependent insulin release from pancreatic beta-cells (2). More recently, liraglutide, a GLP-1A sharing 97% homology with native GLP-1, was approved as an adjunct to lifestyle therapy in obesity management in adults (3).

Weight loss induced by GLP-1A treatment is largely attributed to reduced appetite and energy intake through GLP-1 signaling in the hypothalamus. Data from recent rodent and human studies indicate GLP-1 receptors are expressed in adipose tissue and adipocytes (4–6). Moreover, there is evidence that GLP-1A may also affect energy expenditure by activating brown adipose tissue (BAT) and recruiting beige/brite adipocytes in white adipose tissue (WAT), a process so-called browning of WAT, through several mechanisms. Central administration of liraglutide to mice was shown to increase sympathetic nervous system (SNS) activity, leading to activation BAT thermogenesis (7) and browning of epididymal WAT (7) by decreasing hypothalamic AMPK activity (7). Moreover, peripheral administration of exenatide induced browning of epididymal WAT in mice by upregulating sirtuin 1 expression in adipocytes (8), and peripheral administration of liraglutide activated invariant natural killer (iNKT) cells resident in WAT to increase fibroblast growth factor 21 (FGF21) production, leading to browning of inguinal white fat in mice. Interestingly, in the latter study it was shown that increased peripheral levels of iNKT cells and FGF-21 were observed in humans treated with liraglutide and were positively correlated with the degree of weight loss induced by therapy (9).

The SNS and thyroid hormone (TH) are critical regulators of BAT and beige/brite adipocyte activity and crosstalk to promote an appropriate thermogenic response (10). The interaction between SNS and TH has been best characterized in BAT. In mature brown adipocytes, catecholamines signal through the β3-adrenergic receptor to activate the thermogenic machinery (11) and through the α1-adrenergic receptor to promote intracellular TH activation by increasing type 2 deiodinase (D2) activity (12). On the other hand, TH enhances adrenergic signaling in adipocytes through TH receptor alpha activation (13) and directly increases the expression of thermogenesis-related genes by TH receptor beta-mediated signaling (10). Despite previous evidence indicating that GLP-1A activate thermogenic fat, at least in part, by increasing SNS activity (7), it is unknown whether GLP-1 signaling could affect D2 activity or adrenergic action in adipose tissue.

To further investigate the effects of GLP-1A on the main regulators of the thermogenic response in adipose tissue, we examined the impact of prolonged administration of liraglutide to obese mice on D2 activity in interscapular BAT and inguinal WAT, and on β3-adrenergic-induced UCP-1 expression and oxygen consumption in both adipose tissue depots.

## Methods

### Animal Care and Maintenance

The number of animals required for the study was calculated using *a priori* power analysis, considering weight loss as the main outcome (G*Power 3.1.9.4). In a pilot study, liraglutide (200 or 400 μg/kg body weight/d) or vehicle were administered to 16-week-old male C57BL/6J mice fed a high-fat diet since the age of 5 weeks (n = 5 mice/group), for 14 days (**Supplementary Figure 1**). As expected, liraglutide treatment at both doses reduced fasting blood glucose levels and energy intake, in addition to inducing weight loss and diminishing hepatic fat accumulation. There were no signs of toxicity as indicated by animal observation and hepatic histologic features.

Mice treated with liraglutide at 400 μg/kg body weight/d exhibited a mean loss of 19.2% of initial body weight (-7.10 ± 1.2 g), whereas vehicle-treated mice gained a mean of mean 2.1% gain of initial body weight (+ 0.79 ± 1.17 g),. Setting type I and II errors at 0.05 and the effect size Cohen’s d = 7.6, a sample size of 5 mice per group would provide a power of over 95%.

Male C57BL/6J mice were purchased from the University of Sao Paulo, Brazil, at the age of 4 weeks, and allowed to acclimate to the new environment for 1 week. They were housed under specific pathogen-free conditions, in individually ventilated cages with 5 to 6 mice per cage until the age of 10 weeks, and 1 mouse per cage thereafter. Mice were maintained at controlled temperature (25°C) and illumination (12-h light/dark cycle), with ad libitum access to water/food.

At the end of the experiment, mice were anesthetized with inhalant 5% isoflurane and euthanized by decapitation. Interscapular BAT (iBAT) and inguinal WAT (ingWAT) were removed, and immediately assessed for oxygen consumption or frozen in liquid nitrogen and kept at -80°C until analysis. Experiments were approved by the Ethics Committee of the University of Brasilia.

### Animal Study

**Figure 1:**
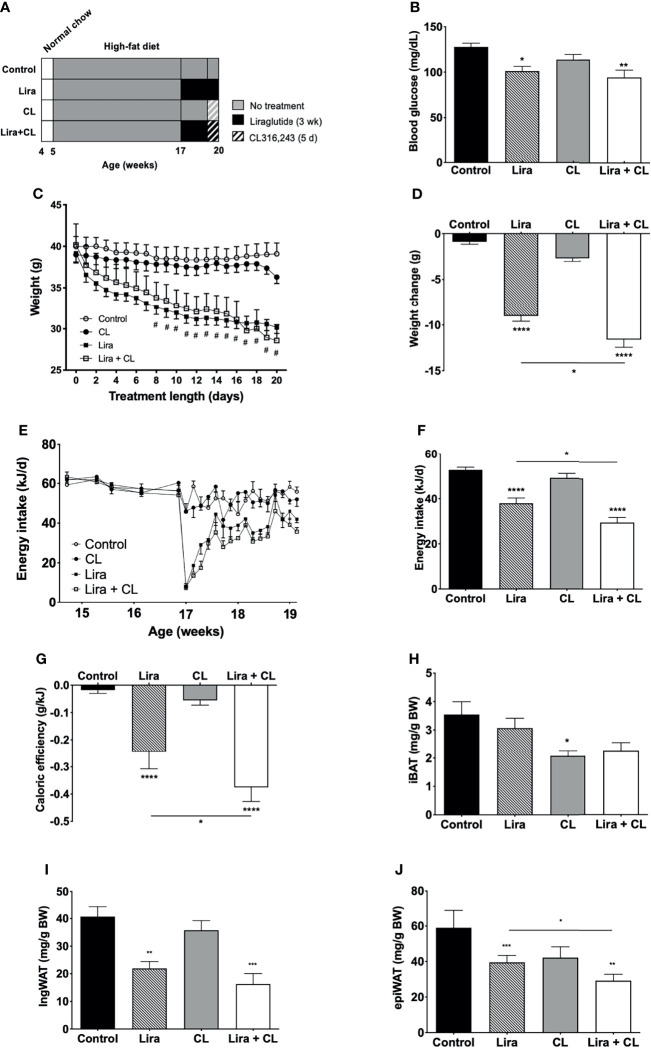
Effect of liraglutide and β3-AR stimulation on fasting blood glucose, body weight, fat mass, and food intake. **(A)** Study design: male C57BL/6J received liraglutide (400 μg/kg/d) or control for 21 days, ± the β3-adrenergic agonist CL 316,243 (CL) during the last 5 days of treatment. **(B)** Fasting blood glucose levels after liraglutide and CL treatment, **(C)** body weight before and during liraglutide and CL treatment, **(D)** body weight change after liraglutide and CL treatment, **(E)** energy intake before and during liraglutide and CL treatment, **(F)** mean daily energy intake during liraglutide and CL treatment, **(G)** caloric efficiency, and **(H)** iBAT, **(I)** ingWAT, and **(J)** epiWAT mass. ^#^p < 0.05 liraglutide-treated mice (Lira and Lira + CL) vs vehicle by Two-way ANOVA followed by Tukey’s multiple comparison test. *p < 0.05, **p < 0.01, ***p < 0.001, ****p < 0.0001 vs control or indicated group, by One-way ANOVA followed by Tukey’s multiple comparison test. Data presented as mean ± SEM, n=5-6 for each experimental group. CL, CL316,243; epiWAT, epidydimal white adipose tissue; iBAT, interscapular brown adipose tissue; ingWAT, inguinal white adipose tissue; Lira, liraglutide.

After the age of 5 weeks, mice were fed a high-fat diet (60% kcal from fat, 20% kcal from protein, and 20% kcal from carbohydrate; D12492, Research Diets, New Brunswik, NK). At 17 weeks, they were randomly assigned to receive intraperitoneal liraglutide (400 μg/kg/d, Novo Nordisk; n = 11) or vehicle (saline; n = 11), for 3 weeks. After 16 days of treatment, five mice in each group were randomly assigned to receive the β3-adrenergic selective agonist CL316,243 (1 mg/kg body weight/d, the last dose administered 2 h before euthanasia; Sigma-Aldrich) or vehicle (saline, **Figure 1A**), using the intraperitoneal route. Both randomizations were stratified according to weight and conducted using Microsoft Excel. Body weight and food intake were measured weekly until 17 weeks, and daily during treatment. Caloric or feeding efficiency was calculated by dividing weight gain by energy intake during treatment. Treatments and outcome assessment were conducted in the morning (8 to 10 am). The individual mouse was considered the experimental unit. Investigators administering treatments (liraglutide, CL316,243, saline) and assessing outcomes were aware of the treatment group allocation, whereas data analysis was conducted by a different researcher in a blinded manner.

### Immunohistochemistry

BAT and WAT immunostaining for UCP-1 was assessed using polyclonal rabbit anti-UCP-1 antibody (1:100, Santa Cruz Biotechnology, sc-6528) and R.T.U Vectastain Universal Quick Kit (Vector Laboratories, PK-7800). Sections were imaged with a digital slide scanner (Leica Biosystems). ingWAT adipocyte diameter was determined using ImageJ, as previously described (14).

### RNA Isolation and Real-Time PCR

Total RNA was isolated from BAT, ingWAT, and epiWAT using Trizol (Invitrogen) and chloroform-isopropanol (Sigma-Aldrich), treated with RNase-free DNase I (Sigma-Aldrich) and used for real-time quantitative PCR with Power SYBR^®^ Green RNA-to-CT™ 1-Step kit (Applied Biosystems). Relative mRNA expression was calculated by the 2^-ΔΔ^ *^C^* ^q^ method using *Gapdh* as the reference gene (**Supplementary Figure 2**). Due to low expression levels in WAT, relative *Ucp1* mRNA expression was calculated by the 2^-Δ^ *^C^* ^q^ method (15). Primer sequences are described in **Supplementary Table 1**, and MIQE checklist is presented in **Supplementary Table 2**.

### Tissue Oxygen Consumption

Total oxygen consumption was measured in fresh iBAT (20-50 mg sample/mouse) and ingWAT (50-100 mg sample/mouse) using a Clark-type electrode (Strathkelvin). Briefly, immediately after euthanasia, fat depots were dissected and placed in sterile saline on ice. Samples were cut into pieces weighed (20 to 50 mg for iBAT and 50-100 mg for ingWAT, from each mouse). The samples were then placed in empty 1.5 mL tubes and thoroughly minced using a spring scissors. Minced samples were then placed into the respirometer chamber containing respiration buffer (2% albumin (w/v), 25 mM glucose and 1.1 mM sodium pyruvate in DPBS), at 37°C. The chamber was closed, and the rate of total oxygen consumption was measured for 15 sec. Total oxygen consumption was normalized to tissue weight and expressed as ug O_2_/min/mg tissue.

### Type 2 Deiodinase Activity

Tissue samples were homogenized and sonicated on ice in PE buffer (0.1 M potassium phosphate, 1 mM EDTA) containing 0.25 M sucrose, and 10 mM dithiothreitol, pH 6.9. The reaction mixtures containing 100-250 μg tissue proteins were incubated with 100,000 c.p.m. of L-[^125^I]T4 (Amersham), 1 nM unlabeled T4, 20 mM DTT, and 1 mM propylthiouracil (Sigma-Aldrich) in PE buffer at 37°C for 2-3 h. Reactions were terminated by the addition of 200 μL horse serum and 100 μL 50% trichloroacetic acid. After centrifugation at 12,000 *g* for 3 min, free ^125^I in the supernatant was counted using a gamma counter. Activity was expressed as femtomoles iodide generated/min per mg protein. All reactions were performed in duplicates or triplicates, and the experiments were repeated twice for each sample from each mouse. Liver samples were assessed as a negative control (16), and no D2 activity was detected (data not shown).

### Data Report and Analysis

Results were reported using the Animal Research: Reporting *In Vivo* Experiments guidelines (17), and data from all mice was included in data presentation and analysis, since there were no losses. Data were presented as mean ± standard error of mean (SEM), and analyses were performed using GraphPad Prism 7.0 (GraphPad). Statistical significance was considered at p < 0.05, by specific tests described in the figure legends.

## Results

### Liraglutide Decreases Body Weight, Body Fat, and Caloric Efficiency

Daily peripheral administration of liraglutide lowered fasting blood glucose levels (**Figure 1B**) and led to persistent weight decrease after eight days (**Figure 1C**), inducing significant weight loss at the end of treatment (**Figure 1D**). Liraglutide treatment also diminished energy intake (**Figures 1E, F**). The reduction was most pronounced acutely after treatment initiation and was partially reversed after six days (**Figure 1E**). Liraglutide also reduced caloric efficiency (**Figure 1G**), in addition to ingWAT and epiWAT mass (**Figures 1H–J**). Body weight, energy intake, caloric efficiency, and epiWAT mass were further reduced by short-term treatment with CL316,243 in liraglutide-treated mice (**Figures 1D–H**). GLP-1 receptor mRNA was not detected in any of the adipose depots assessed (iBAT, ingWAT, and epiWAT; data not shown). Arcuate nucleus samples were used as positive controls and exhibited detectable levels of GLP-1 receptor mRNA (mean Cq value of 25.7).

### Liraglutide Activates BAT, Induces Browning of WAT, and Enhances β3-Adrenergic-Induced Adipose Tissue Oxygen Consumption

**Figure 2:**
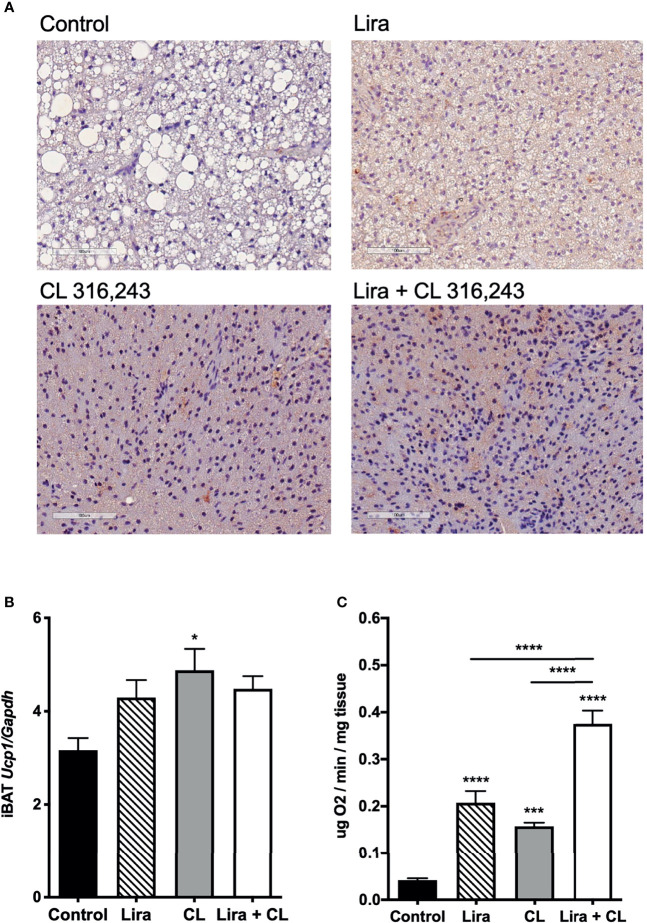
Additive effects of liraglutide and β3-AR stimulation in activating iBAT. **(A)** iBAT immunostaining for UCP-1 (magnification: X10; scale bar: 100 μm), **(B)** iBAT UCP-1 mRNA levels, **(C)** iBAT oxygen consumption. *p < 0.05, ***p < 0.001, ****p < 0.0001 vs control or indicated group, by One-way ANOVA followed by Tukey’s multiple comparison test. Data presented as mean ± SEM, n=5-6 for each experimental group. CL, CL316,243; iBAT, interscapular brown adipose tissue; Lira, liraglutide.

Prolonged peripheral administration of liraglutide reduced lipid droplet size and upregulated UCP-1 protein expression in iBAT (**Figure 2A**). However, UCP-1 mRNA levels assessed at the end of treatment were unchanged in iBAT of liraglutide-treated mice compared with control (**Figure 2B**). Accordingly, iBAT from liraglutide-mice exhibited increased oxygen consumption (**Figure 2C**).

β3-adrenergic stimulation by CL316,243 treatment for five days upregulated UCP-1 mRNA and protein expression in iBAT (**Figures 2A, B**), in addition to increasing oxygen consumption at this depot (**Figure 2C**). Interestingly, liraglutide-induced UCP-1 protein expression and oxygen consumption were enhanced by short-term β3-adrenergic stimulation (**Figures 2A, C**).

**Figure 3:**
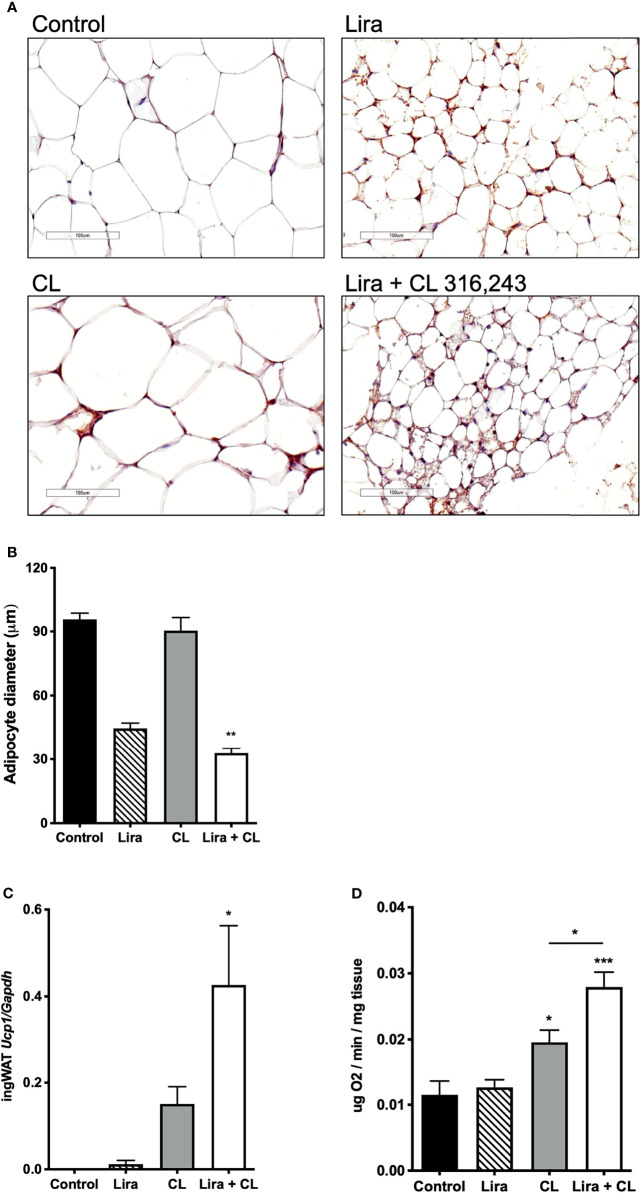
Additive effects of liraglutide and β3-AR stimulation in inducing browning of ingWAT. **(A)** ingWAT immunostaining for UCP-1 (magnification: X10; scale bar: 200 μm), **(B)** adipocyte diameter, **(C)** ingWAT UCP-1 mRNA levels, **(D)** ingWAT oxygen consumption. *p < 0.05, **p < 0.001, ***p < 0.001, vs control or indicated group, by One-way ANOVA followed by Tukey’s multiple comparison test. Data presented as mean ± SEM, n=5-6 for each experimental group. CL, CL316,243; ingWAT, inguinal white adipose tissue; Lira, liraglutide.

We also assessed the effect of prolonged liraglutide treatment on ingWAT. Liraglutide reduced lipid droplet size and induced the appearance of multilocular adipocytes in ingWAT (**Figures 3A, B**), a feature of beige/brite adipocytes (18). Moreover, liraglutide upregulated UCP-1 protein (**Figure 3A**) but not UCP-1 mRNA (**Figure 3C**) expression in ingWAT, assessed at the end of treatment. Oxygen consumption was unchanged in ingWAT by treatment with liraglutide alone compared with control (**Figure 3D**).

Short-term administration of the β3-adrenergic agonist CL316,243 did not affect ingWAT adipocyte size (**Figure 3A**) but upregulated UCP-1 mRNA expression (**Figure 3B**) and increased oxygen consumption (**Figure 3C**). In mice treated with liraglutide, short-term β3-adrenergic stimulation significantly upregulated UCP-1 protein (**Figure 3A**) and mRNA expression (**Figure 3B**) compared with either treatment alone. Moreover, oxygen consumption was significantly increased by co-treatment with liraglutide and CL316,243, compared with CL316,243 alone (**Figure 3C**).

Prolonged liraglutide treatment did not change UCP-1 mRNA levels in epiWAT, but increased UCP-1 protein content. Short term administration of CL316,243 increased UCP-1 mRNA and protein levels in epiWAT. In mice treated with liraglutide, CL316,243 enhanced UCP-1 protein expression compared with liraglutide treatment alone (**Supplementary Figure 4**).

### Liraglutide Increases Type 2 Deiodinase Activity in iBAT

**Figure 4:**
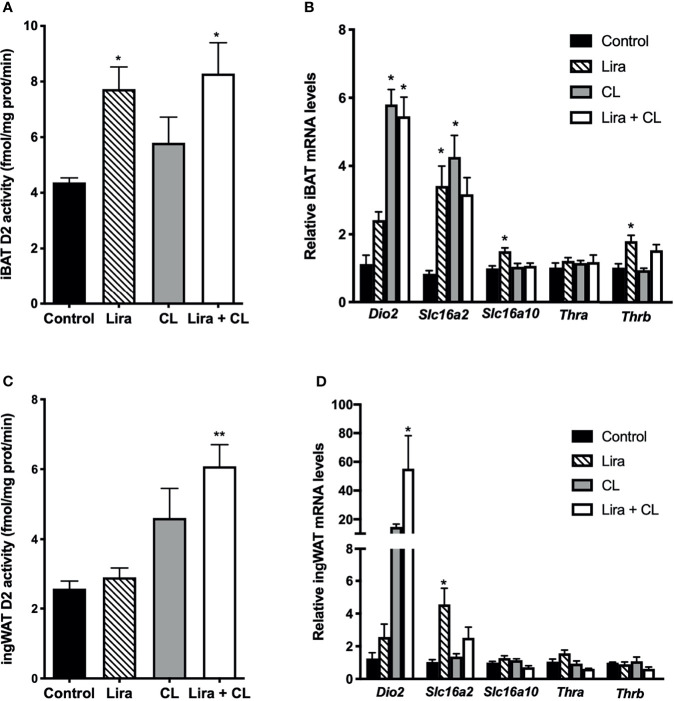
Effect of liraglutide treatment on D2 activity in adipose tissue. **(A)** D2 activity in iBAT, **(B)** mRNA levels of D2, thyroid hormone transporters and thyroid hormone receptors in iBAT, **(C)** D2 activity in ingWAT, **(D)** mRNA levels of D2, thyroid hormone transporters and thyroid hormone receptors in ingWAT. *p < 0.05, **p < 0.01 vs control by One-way ANOVA followed by Tukey’s multiple comparison test. Data presented as mean ± SEM, n=5-6 for each experimental group. CL, CL316,243; iBAT, interscapular brown adipose tissue; ingWAT, inguinal white adipose tissue; Lira, liraglutide.

Given the critical role of intracellular TH activation by D2 in thermogenic adipocytes (19), we investigated whether liraglutide treatment affected D2 mRNA expression and activity. D2 activity was significantly higher in iBAT of vehicle-treated mice when compared with WAT (**Supplementary Figure 3**). Prolonged liraglutide administration significantly upregulated D2 activity in iBAT (**Figure 4A**), despite not changing D2 mRNA levels (**Figure 4B**). Short-term β3-adrenergic stimulation with CL316,243 did not change D2 activity (**Figure 4A**), despite inducing D2 mRNA expression in iBAT (**Figure 4B**). Moreover, short-term β3-adrenergic stimulation did not affect liraglutide-induced upregulation of D2 activity (**Figure 4A**). We also found that both liraglutide and β3-adrenergic activation increased SLC16A2 mRNA levels, and liraglutide slightly induced THRB mRNA levels (**Figure 4B**).

Liraglutide did not change D2 activity in ingWAT (**Figure 4C**), despite significantly inducing D2 mRNA levels (**Figure 4D**). However, liraglutide treatment enhanced β3-adrenergic signaling-induced upregulation of D2 activity (**Figure 4C**). Moreover, liraglutide induced SLC16A2 mRNA expression in ingWAT (**Figure 4D**).

In epiWAT, liraglutide treatment did not modify D2 mRNA levels or D2 activity, but upregulated SLC16A2 mRNA expression. Short-term β3-adrenergic agonist treatment increased D2 mRNA levels and did not affect D2 activity (**Supplementary Figure 4**).

## Discussion

We report that prolonged peripheral administration of liraglutide increases D2 activity in BAT of obese male mice to a greater extent than β3-adrenergic stimulation. To our knowledge, this is the first evidence suggesting that liraglutide may modulate brown fat function by inducing D2 activity, which acts to promote intracellular TH activation. In addition, we observed that liraglutide enhanced UCP-1 upregulation and oxygen consumption induced by β3-adrenergic signaling in both BAT and subcutaneous WAT, a finding that could suggest that liraglutide affects thermogenic fat by mechanisms other than SNS activation.

Liraglutide treatment reduced body weight, body fat, and energy intake of male obese mice, in agreement with previous data from human (20) and rodent (21) studies. In addition, liraglutide-treated mice showed persistent weight loss despite reduced suppression of food intake after the first week of treatment, suggesting that long-term weight loss induced by GLP-1A is not fully explained by reduced energy intake. Alternative explanations for continued weight loss in this setting are fecal energy loss or increased energy expenditure.

Little is known about how GLP-1 signaling affects fecal energy loss. In humans, it was reported that GLP-1 (22) or liraglutide (23) administration diminished small intestine motility leading to increased transit time in subjects with normal glucose tolerance (22) or type 2 diabetes (23). Moreover, GLP-1 receptor signaling by endogenous GLP-1 or exogenous agonists exhibited intestinotrophic actions in mice, mediated by fibroblast growth factor 7 (24). Despite potentially augmenting nutrient absorption, whether GLP-1 receptor-mediated actions to increase small intestine transit time or promote intestinal growth affect fecal energy loss remains unexplored. Additionally, in mouse models of obesity, liraglutide treatment induced significant gut microbiome changes (25). Although it is not possible to rule out that the latter changes were secondary to liraglutide-induced weight loss per se, it is reasonable to hypothesize that gut microbiome composition modifications could affect nutrient absorption and, therefore, fecal energy loss.

An alternative explanation for continued weight loss induced by liraglutide despite a less pronounced reduction in food intake with prolonged treatment is increased energy expenditure. The latter is supported by evidence indicating that GLP-1 signaling affects energy expenditure in animal models (7, 21) by increasing the sympathetic output to thermogenic fat (7). Notably, energy expenditure changes in response to GLP-1A treatment were also assessed in human studies. It was reported that obese subjects with type 2 diabetes treated liraglutide or exenatide for 52 weeks showed a significant decrease in body mass index and total fat mass and increase in fat-free mass (7). These findings were accompanied by increased resting energy expenditure adjusted for fat-free mass (7). Accordingly, a longitudinal study revealed that 12-week exenatide treatment increased BAT activity measured by [^18^F]fluorodeoxyglucose positron emission tomography (26).

Although we did not assess energy expenditure, we observed a significant decrease in the weight gain-caloric intake ratio (caloric efficiency) of liraglutide-treated mice. This finding would not be expected in the setting of weight loss induced solely by diminished caloric intake. It should be pointed out that caloric efficiency is most accurately assessed when caloric output in feces is taken into account to determine the net energy input. However, weight loss due to caloric restriction is expected to decrease caloric output in feces (27). Therefore, it is most likely that we underestimated caloric input and the reduction in caloric efficiency.

Unlike previous studies that assessed whole-body energy expenditure (7, 21), we examined oxygen consumption directly in adipose tissue and found that liraglutide increased oxygen consumption to levels comparable to those induced by β3-adrenergic signaling. Increased total oxygen consumption in BAT and WAT, in the setting of weight loss and reduced caloric efficiency, most likely reflects increased thermogenesis. Indeed, weight reduction solely due to diminished energy intake reduces energy expenditure in animal models (28) and humans (29, 30). Therefore, it would be expected to diminish adipose tissue oxygen consumption. Moreover, the increased adipose tissue oxygen consumption found herein was accompanied by upregulation of UCP-1 protein levels in iBAT and ingWAT, indicating BAT activation and browning of WAT (18). The latter findings are also in agreement with increased thermogenesis.

It is important to point that liraglutide did not significantly change iBAT UCP-1 mRNA levels, despite increasing UCP-1 protein expression and oxygen consumption in this adipose depot. This is consistent with previous data indicating that UCP-1 mRNA expression does not parallel BAT thermogenic activity (31, 32). Moreover, the higher fold-induction of UCP-1 mRNA in ingWAT of liraglutide- and CL316,243-treated mice, compared with UCP-1 protein and oxygen consumption induction, is also in accordance with previous findings and most likely stems from lower expression levels in unstimulated WAT (32).

Interestingly, liraglutide enhanced β3-adrenergic-induced oxygen consumption in both iBAT and ingWAT. UCP-1 protein levels in iBAT and ingWAT were also additively upregulated by liraglutide and β3-adrenergic stimulation. This finding may reflect nonsympathetically-mediated action of liraglutide on thermogenic fat since additive effects to β3-adrenergic stimulation would not be expected if liraglutide promoted oxygen consumption exclusively by SNS activation. The dose of the β3-adrenergic agonist used herein, CL316,243, is presumably a saturating one (33). Hence, it would be unlikely to observe the effect of sympathetic activation in the setting of maximal β3-adrenergic activation.

Nonsympathetically-mediated actions of liraglutide on thermogenic fat could be explained by direct signaling on adipose tissue. Indeed, data from cell-based studies indicate direct effects of GLP-1 on adipocytes, such as adipogenesis induction (5) and activation of both lipogenesis and lipolysis dependent upon GLP-1/GLP-1A concentration (34, 35). Importantly, 3T3-L1 adipocyte treatment with exendin-4 increased mitochondrial biogenesis and function (8). Similarly, liraglutide-treated 3T3-L1 adipocytes exhibited a thermogenic phenotype, indicated by increased expression of thermogenesis-related genes and proteins, in addition to increased mitochondrial biogenesis (36). Although our study design precludes defining direct actions liraglutide on adipose tissue, the latter findings from cell-based studies support our hypothesis that additive effects of liraglutide and maximal β3-adrenergic activation on iBAT and ingWAT could suggest that liraglutide can affect adipose tissue by mechanisms other than sympathetic activation, such as direct signaling in adipose tissue. An additional line of evidence supporting the possibility of a nonsympathetically-mediated action of liraglutide on adipose tissue found herein is the previous report that GLP-1-induced activation of SNS is blunted in obese mice (37).

GLP-1 mRNA was not detected in any of the adipose tissue depots assessed herein, although GLP-1 receptor protein expression was previously shown in both mouse and human adipose tissue/adipocytes (4–6), as indicated by antibody-based detection methods. However, it is acknowledged that available antibodies against GLP-1 receptor have several limitations with respect to accuracy, reliability, and reproducibility (38–40). Moreover, it is still unclear whether GLP-1 exerts its direct actions on adipose tissue through the ‘classical’ GLP-1 receptor, which signal through the adenylate cyclase/cAMP pathway, or ‘alternative’ GLP-1 receptors, which signal through other pathways (41). It was previously reported that the lipolytic action of GLP-1 in 3T3-L1 adipocytes was accompanied by increased intracellular cAMP levels, implying that the classical receptor was activated by GLP-1 signaling in adipocytes (4). Accordingly, it was shown that the GLP-1A exendin-4 activated adenylate cyclase/cAMP/protein kinase A pathway to induce the expression of adiponectin and suppress inflammatory cytokine expression in 3T3-L1 adipocytes (42).

The possibility of direct GLP-1 actions in adipose tissue mediated by the classical GLP-1 receptor remains elusive. However, it should be pointed that the adenylate cyclase/cAMP/protein kinase A mediates the action of adrenergic signaling and also of several other activators of thermogenic adipocytes, such as adenosine (43), the extracellular matrix protein Slit2 (44), transient receptor potential melastatin 8 (45), and bile acids. The latter were reported to increase energy expenditure by activating G-protein-coupled receptor TGR5/adenylate cyclase/cAMP/protein kinase A signaling pathway on brown adipocytes, leading to increased D2 activity and intracellular TH activation (46). TH is a critical regulator of thermogenic fat activity (19, 47), acting synergistically with the SNS (48). Intracellular TH action in brown adipocytes is determined by the availability of cellular T3, which depends on D2 activity (19).

Given the abovementioned data indicating that the effects of GLP-1 in adipocytes are mediated by the adenylate cyclase/cAMP/protein kinase A signaling pathway activated by G-protein coupled receptors, we investigated whether liraglutide action in adipose tissue to promote increased oxygen consumption could involve TH activation by affecting D2 activity. We found that liraglutide, but not β3-adrenergic stimulation, increased D2 activity in iBAT, possibly through a nontranscriptional mechanism, since D2 mRNA levels were not significantly changed. Interestingly, liraglutide treatment did not affect D2 activity in ingWAT, implying that it may promote TH activation in thermogenic fat in a depot-specific manner. Liraglutide also increased the transcription of the gene encoding the TH transporter SLC16A2 in adipose tissue. Given that the latter mediates TH entry into cells (49), this finding suggests another possible effect to increase intracellular TH action.

In summary, our findings indicate that liraglutide increases D2 activity and oxygen consumption in BAT from obese mice and may, therefore, activate BAT by promoting intracellular TH activation. Moreover, liraglutide enhances the effect of β3-adrenergic stimulation to increase oxygen consumption and UCP-1 expression in both ingWAT and BAT, suggesting it may affect thermogenic fat by mechanisms other than SNS activation. These findings may be clinically relevant and add to the currently known mechanisms of GLP-1A-induced weight loss.

## Data Availability Statement

The original contributions presented in the study are included in the article/**Supplementary Material**. Further inquiries can be directed to the corresponding author.

## Ethics Statement

The animal study was reviewed and approved by Ethics Committee of the University of Brasilia.

## Author Contributions

CR, EB, and SP contributed to data acquisition. FO, SW, and AM contributed to data acquisition, analysis, and interpretation, and writing of the manuscript. MC, FN, and AA contributed to the study concept and design, data analysis and interpretation, and writing of the manuscript. FO and AA are the guarantors of this work and, as such, had full access to all the data in the study and take responsibility for the integrity of the data and the accuracy of the analysis. All authors contributed to the article and approved the submitted version.

## Funding

This work was founded by the National Council on Scientific and Technological Development (grant 420562/2016-8), and by the University of Brasilia (grant DPI 01/2021 and DPI 03/2021). The study sponsor/funder was not involved in the design of the study; the collection, analysis, and interpretation of data; writing the report; and did not impose any restrictions regarding the publication of the report.

## Conflict of Interest

The authors declare that the research was conducted in the absence of any commercial or financial relationships that could be construed as a potential conflict of interest.

## Publisher’s Note

All claims expressed in this article are solely those of the authors and do not necessarily represent those of their affiliated organizations, or those of the publisher, the editors and the reviewers. Any product that may be evaluated in this article, or claim that may be made by its manufacturer, is not guaranteed or endorsed by the publisher.
