# Supplementary material for: Liraglutide Activates Type 2 Deiodinase and Enhances β3-Adrenergic-Induced Thermogenesis in Mouse Adipose Tissue
Source: Front Endocrinol (Lausanne). 2022 Jan 4;12:803363. doi: 10.3389/fendo.2021.803363 (PMC8771968; doi:10.3389/fendo.2021.803363)
Supplement: Supplementary file 1 [file DataSheet_1.docx]

Supplementary Material

**Supplementary Table 1.** Primer sequences used in real-time PCR

| **Gene** | **Forward primer (3’-5’)** | **Reverse primer (3’-5’)** |
| --- | --- | --- |
| *Ucp1* | ACTGCCACACCTCCAGTCATT | CTTTGCCTCACTCAGGATTGG |
| *Dio2* | CAGCTTCCTCCTAGATGCCTA | CTGATTCAGGATTGGAGACGTG |
| *Slc16a2* | CGGCTGGATAGTGGTGTTTG | CAGAGTTATGGATGCCGAAGATG |
| *Slc16a10* | AGGTGCTCTTCATGTGCATTG | TGGAGGTAGACCTTCTTCACAC |
| *Thra* | CTGACCTCCGCATGATCGG | GGTGGGGCACTCGACTTTC |
| *Thrb* | ACACCAGCAATTACCAGAGTG | GCAGCTCGAAGGGACATGA |
| *Glp1r* | ACGGTGTCCCTCTCAGAGAC | ATCAAAGGTCCGGTTGCAGAA |
| *Gapdh* | AAGGGCTCATGACCACAGTC | CAGGGATGATGTTCTGGGCA |

**Supplementary Table 2.** MIQE checklist (essential information)

| **Item** | **Description** |
| --- | --- |
| **Experimental design** | |
| Definition of experimental and control groups | Control group: vehicle-treated mice  Experimental groups: mice treated with (i) liraglutide 400 μg/kg/d (D1-D21), (ii) CL316,243 1 mg/kg/d (D17-D21), or (iii) liraglutide 400 μg/kg/d (D1-D21) and CL316,243 1 mg/kg/d (D17-D21)  qPCR controls (Power SYBR Green RNA-to-C_T_ 1-Step kit):  Negative controls: DPEC-treated water instead of RNA sample.  Control for genomic DNA contamination: SYBR green and DNase I-treated sample without reverse transcriptase. |
| Number within each group | Control group: n = 6 mice  Mice treated with liraglutide 400 μg/kg/d (D1-D21): n = 6 mice  Mice treated with CL316,243 1 mg/kg/d (D17-D21): n = 5  Mice treated with liraglutide 400 μg/kg/d (D1-D21) and CL316,243 1 mg/kg/d (D17-D21): n = 5 |
| **Sample** | |
| Description | Interscapular brown adipose tissue, inguinal white adipose tissue, and epididymal white adipose tissue; tissue samples were dissected and immediately frozen in dry ice. Then, they were kept at -80^o^C until RNA isolation. |
| Microdissection or microdissection | Macrodissection of adipose tissue depots (interscapular brown adipose tissue, inguinal white adipose tissue, epididymal white adipose tissue). |
| Processing procedure | Tissue samples were dissected, placed at RNase free 1.5 mL tubes (Axygen), and immediately frozen in dry ice. Then, they were kept at -80^o^C until RNA isolation. |
| If frozen, how quickly? | Tissue samples were immediately placed in a container with liquid nitrogen. |
| If fixed, with what and how quickly? | Tissue samples were not fixed for real-time PCR assays. |
| Sample storage conditions and duration | Tissue samples were stored at -80^o^C for 6 weeks before RNA isolation. |
| **Nucleic acid extraction** | |
| Procedure and/or instrumentation | RNA was isolated using Trizol (Invitrogen) and chloroform-isopropanol (Sigma-Aldrich), washed with ethanol (Sigma-Aldrich), and solubilized in DEPC-treated water. Purity and concentration were assessed in a spectrophotometer (Nanovue, GE) and only samples with A260/A280 between 1.8 and 2.1 were used. RNA integrity was assessed by documentation of ribosomal bands (28S and 18S) after 1% agarose gel electrophoresis. It was then treated with DNase I (Sigma-Aldrich). Material used for RNA isolation, such as tubes and pipette tips, were RNase free. The environment (bench, racks, and pipettes) was cleaned with 0.2 N NaOH and 0.2% SDS solution. |
| Name of kit and details of any modifications | We used reagents from the following companies: Trizol (Invitrogen), chloroform (Sigma-Aldrich), isopropanol (Sigma-Aldrich), ethanol (Sigma-Aldrich). DNase I kit was obtained from Sigma-Aldrich, and no modification was made to the manufacturer´s protocol. |
| Details of RNase or DNase treatment | The environment (bench) and material (pipettes and racks) were treated with wiped with 0.2 N NaOH and 0.2% SDS solution.  All tubes and pipette tips were RNase free (obtained from Axygen).  DNase treatment was conducted with DNase I kit from Sigma-Aldrich, following the manufacturer’s instructions. |
| Contamination assessment (RNA) | Conducted on a spectrophotometer (Nanovue, GE).  Only samples with A260/A280 between 1.8 and 2.1 were used. |
| Nucleic acid quantification. | Conducted on a spectrophotometer (Nanovue, GE).  Calculated from measured absorbance values at 260 nm, using the Beer-Lambert’s equation. |
| Instrument and method | Contamination assessment and RNA quantification were conducted using a spectrophotometer (Nanovue, GE), automatically by the equipment (calculation of A260/A280 ratio for contamination assessment and using Beer-Lambert’s equation for quantification). |
| RNA integrity | RNA integrity was defined by the observation of ribosomal bands (28S and 18S) after 1% agarose gel electrophoresis. |
| RIN/RQI or Cq of 3′ and 5′ transcripts | Not assessed. |
| Inhibition testing (Cq dilutions, spike, or other) | Not conducted. |
| **Reverse transcription** | |
| Complete reaction conditions | Reverse transcription and qPCR were conducted using Power SYBR Green RNA-to-C_T_ 1-Step kit (Thermofisher, Catalog Number 4389986), according to the manufacturer’s instructions. The reaction conditions were as follows:   \| **Stage** \| **Step** \| **Temperature** \| **Time** \| \| --- \| --- \| --- \| --- \| \| Holding \| Reverse transcription \| 48^o^C \| 30 min \| \| Holding \| Activation of AmpliTaq Gold DNA Polimerase, UP (Ultra-Pure) \| 95^o^C \| 10 min \| \| Cycling  (40 cycles) \| Denature \| 95^o^C \| 15 sec \| \|  \| Anneal-Extend \| 60^o^C \| 1 min \| \| Melt curve \| Denature \| 95^o^C \| 15 sec \| \|  \| Anneal \| 60^o^C \| 15 sec \| \|  \| Denature \| 95^o^C \| 15 sec \| |
| Amount of RNA and reaction volume | 5 ng of RNA per reaction; reaction volume: 10 μL. |
| Priming oligonucleotide and concentration | Data not provided by the manufacturer (Power SYBR Green RNA-to-C_T_ 1-Step kit, Thermofisher, Catalog Number 4389986). |
| Reverse transcriptase concentration | Data not provided by the manufacturer (Power SYBR Green RNA-to-C_T_ 1-Step kit, Thermofisher, Catalog Number 4389986). |
| Temperature and time | 48^o^C, 30 min (as described by the manufacturer - Power SYBR Green RNA-to-C_T_ 1-Step kit, Thermofisher, Catalog Number 4389986). |
| **qPCR target information** | |
| Gene symbol | Ucp1, Dio2, Slc16a2, Slc16a10, Thra, Thrb, Glp1r, Gapdh |
| Sequence accession number | Ucp1: AC122890.4  Dio2: AH009875.2  Slc16a2: AC164176.12  Slc16a10: [AL360227.17](https://www.ncbi.nlm.nih.gov/nuccore/10443098)  Thra: AL590963.11  Thrb: [AC138400.12](https://www.ncbi.nlm.nih.gov/nuccore/39930748)  Glp1r: AC165951.3  Gapdh: [AC166162.6](https://www.ncbi.nlm.nih.gov/nuccore/78190259) |
| Amplicon length | Ucp1: 123 base pairs  Dio2: 89 base pairs  Slc16a2: 65 base pairs  Slc16a10: 100 base pairs  Thra: 68 base pairs  Thrb: 125 base pairs  Glp1r: 117 base pairs  Gapdh: 111 base pairs |
| In silico specificity screen (BLAST, and so on) | Ucp1:  https://www.ncbi.nlm.nih.gov/tools/primer-blast/primertool.cgi?ctg_time=1637102796&job_key=4Oo_9MnxxFnjZ95i0wL6UKkZ62KECvB_hQ  Dio2:  https://www.ncbi.nlm.nih.gov/tools/primer-blast/primertool.cgi?ctg_time=1637103305&job_key=uLJnrJHxnFm7Z4ZiiwKiUPEZs2LcCqh_3Q  Slc16a2:  https://www.ncbi.nlm.nih.gov/tools/primer-blast/primertool.cgi?ctg_time=1637110526&job_key=BA7bhAvIBmAhXgNbDjsnaXQgNltZMy1GWA  Slc16a10:  https://www.ncbi.nlm.nih.gov/tools/primer-blast/primertool.cgi?ctg_time=1637110586&job_key=6eM2aNJt38X4_0_6QppryDiBevoVkmHnFA  Thra:  https://www.ncbi.nlm.nih.gov/tools/primer-blast/primertool.cgi?ctg_time=1637110699&job_key=w8kc1-_04lzFYvhn9QfcVY8czWeiD9Z6ow  Thrb:  https://www.ncbi.nlm.nih.gov/tools/primer-blast/primertool.cgi?ctg_time=1637110721&job_key=gYteALptt8WQ_yf6KpoDyFCBEvp9kgnnfA  Glp1r:  https://www.ncbi.nlm.nih.gov/tools/primer-blast/primertool.cgi?ctg_time=1639258506&job_key=AgjdEA3XAH8nQRpEFyQ-dm0_L0RALDRZQQ  Gapdh:  https://www.ncbi.nlm.nih.gov/tools/primer-blast/primertool.cgi?ctg_time=1637110917&job_key=-fMm7dX02Fz_YsJnzwfmVbUc92eYD-x6mQ |
| Primer validation | \| Primer pair \| Sample for validation \| Slope \| R^2^ \| Efficiency (%) \| \| --- \| --- \| --- \| --- \| --- \| \| *Ucp1* \| BAT \| -3.167 \| 0.994 \| 106.88 \| \| *Dio2* \| BAT \| -3.349 \| 0.997 \| 98.88 \| \| *Slc16a2* \| Liver \| -3.104 \| 0.988 \| 109.90 \| \| *Slc16a10* \| Liver \| -3.342 \| 0.988 \| 99.17 \| \| *Thra* \| Heart \| -3.2 \| 0.986 \| 105.29 \| \| *Thrb* \| Liver \| -3.5 \| 0.982 \| 93.01 \| \| *Glp1R* \| Arcuate nucleus \| -3.068 \| 0.993 \| 111.83 \| \| *Gapdh* \| Epididymal WAT \| -3.3 \| 0.980 \| 100.81 \| |

**A**

**C**

**D**


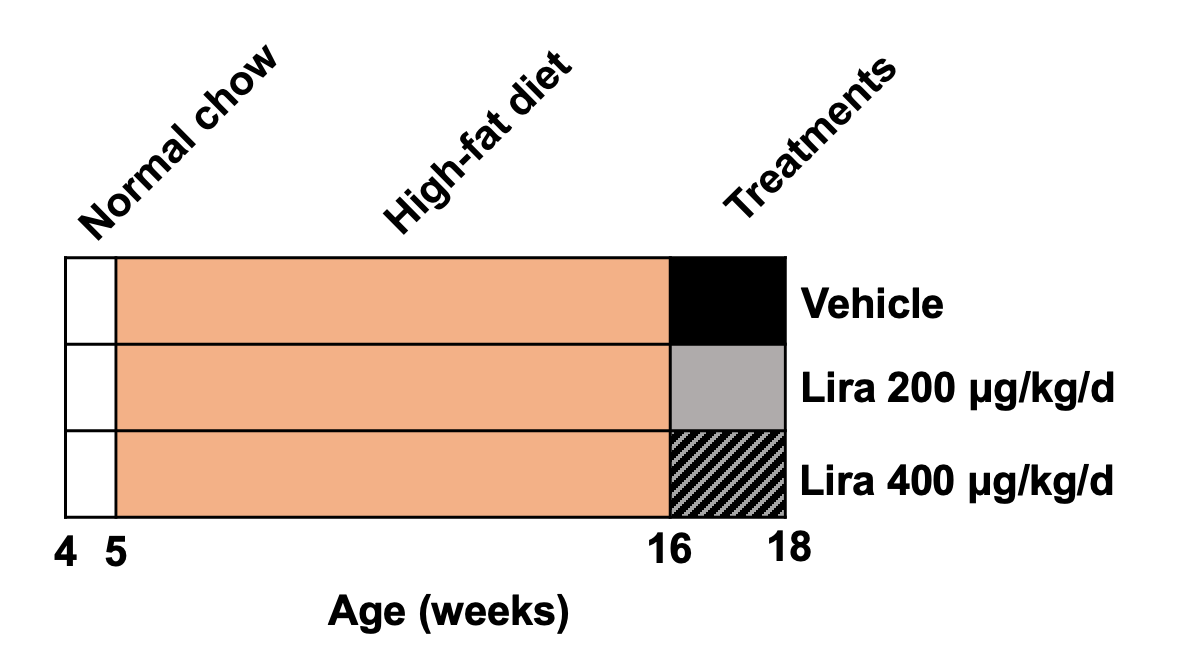


**E**

**F**

**B**

**G**


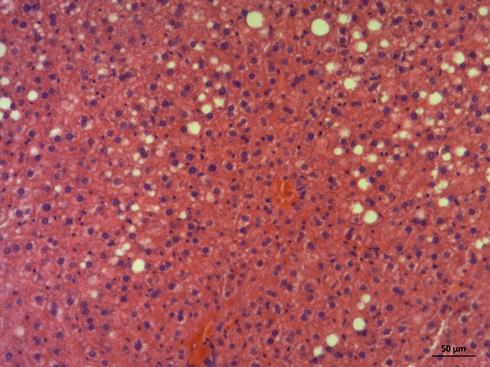

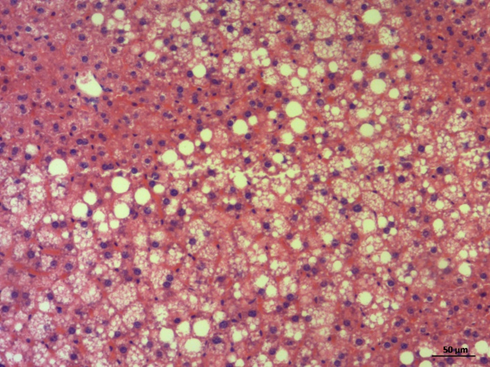

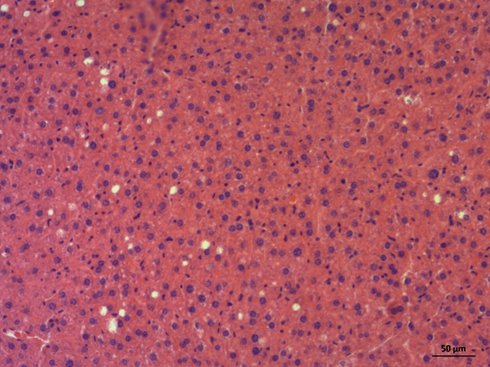


Vehicle

Lira 200 μg/kg/d

Lira 400 μg/kg/d

**Supplementary Figure 1.** Effect of liraglutide on fasting blood glucose levels, body weight, food intake, and liver histology. (A) Study design: male C57BL/6J received liraglutide (200 or 400 μg/kg/d) or vehicle control for 14 days. (B) Fasting blood glucose levels after treatment, (C) body weight before and treatment, (D) body weight change after treatment, (E) mean daily energy intake during treatment, (G) caloric efficiency during treatment, and (F) liver histology after treatment. * p < 0.05, **** p < 0.0001 vs control group, by One-way ANOVA followed by Tukey’s multiple comparison test. Data presented as mean ± SEM, n=5 for each experimental group. Lira: liraglutide.

**Supplementary Figure 2.** Mean Ct values from PCRs for reference gene (*Gapdh*) in different adipose tissue depots. One-way ANOVA followed by Tukey’s multiple comparison test. Data presented as mean ± SEM, n=5-6 for each experimental group. CL: CL316,243; epiWAT: epidydimal white adipose tissue; iBAT: interscapular brown adipose tissue; ingWAT: inguinal white adipose tissue; Lira: liraglutide.

**Supplementary Figure 3.** D2 activity in different adipose tissue depots of vehicle-treated mice fed a high-fat diet. *** p < 0.001 vs iBAT by One-way ANOVA followed by Tukey’s multiple comparison test. Data presented as mean ± SEM, n=5-6 for each experimental group. CL: CL316,243; epiWAT: epidydimal white adipose tissue; iBAT: interscapular brown adipose tissue; ingWAT: inguinal white adipose tissue; Lira: liraglutide.

Control

Lira

CL 316,243

Lira + CL 316,243

**A**

**B**

**C**


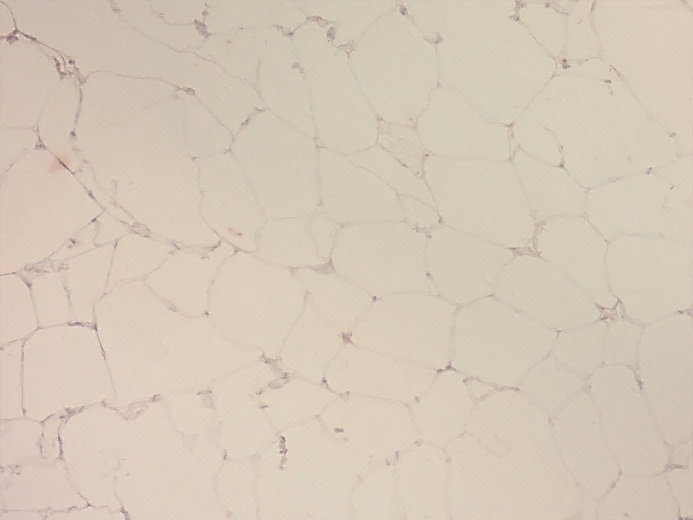

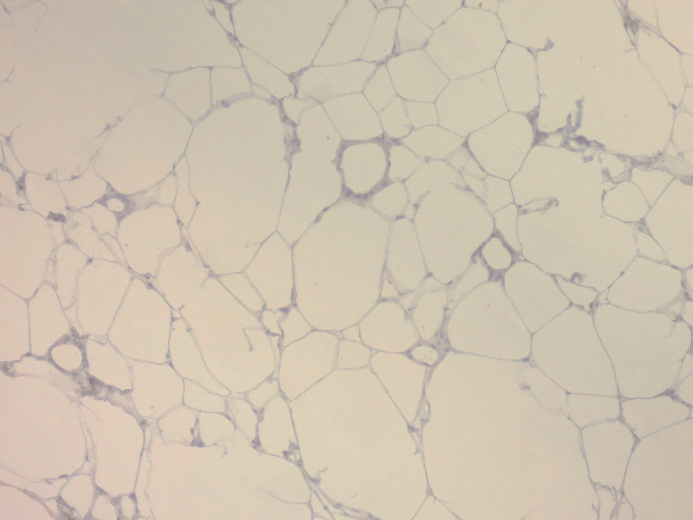

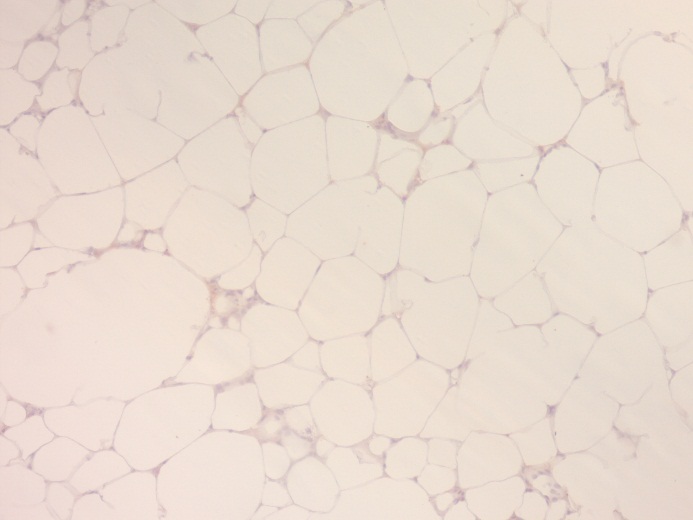

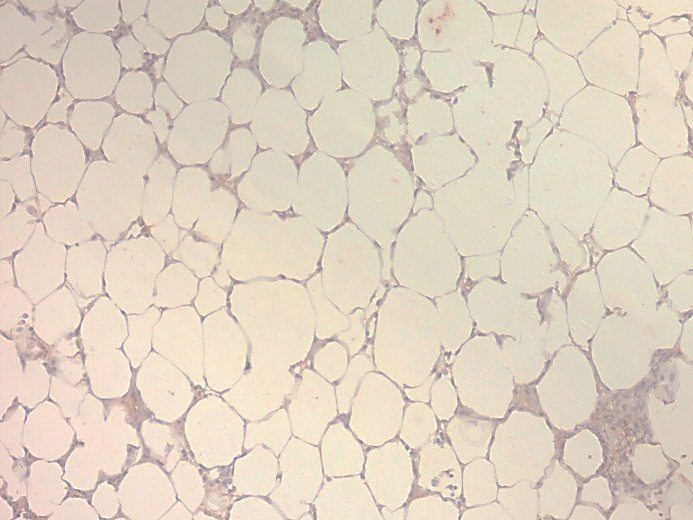


**D**

**Supplementary Figure 4.** Effects of liraglutide and β3-AR stimulation in epiWAT.

(A) epiWAT immunostaining for UCP-1 (magnification: X20; scale bar: 100 μm), (B) epiWAT UCP-1 mRNA levels, (C) D2 activity in epiWAT, (D) mRNA levels of D2, thyroid hormone transporters and thyroid hormone receptors in epiWAT. * p < 0.05 vs control (vehicle-treated mice) by One-way ANOVA followed by Tukey’s multiple comparison test. Data presented as mean ± SEM, n=5-6 for each experimental group.
